# NanoHIV: A Bioinformatics Pipeline for Producing Accurate, Near Full-Length HIV Proviral Genomes Sequenced Using the Oxford Nanopore Technology

**DOI:** 10.3390/cells10102577

**Published:** 2021-09-28

**Authors:** Imogen A. Wright, Kayla E. Delaney, Mary Grace K. Katusiime, Johannes C. Botha, Susan Engelbrecht, Mary F. Kearney, Gert U. van Zyl

**Affiliations:** 1 Division of Medical Virology, Stellenbosch University and NHLS Tygerberg, Cape Town 7505, South Africa; 18426395@sun.ac.za (K.E.D.); bothajc@sun.ac.za (J.C.B.); susanen@sun.ac.za (S.E.); guvz@sun.ac.za (G.U.v.Z.); 2 HIV Dynamic and Replication Program, Center for Cancer Research, National Cancer Institute at Frederick, 1050 Boyles Street, Building 535, Room 109, Frederick, MD 21702-1201, USA; marygrace.katusiime@nih.gov (M.G.K.K.); kearneym@mail.nih.gov (M.F.K.)

**Keywords:** HIV, nanopore, proviral, consensus, single-genome

## Abstract

HIV-1 proviral single-genome sequencing by limiting-dilution polymerase chain reaction (PCR) amplification is important for differentiating the sequence-intact from defective proviruses that persist during antiretroviral therapy (ART). Intact proviruses may rebound if ART is interrupted and are the barrier to an HIV cure. Oxford Nanopore Technologies (ONT) sequencing offers a promising, cost-effective approach to the sequencing of long amplicons such as near full-length HIV-1 proviruses, but the high diversity of HIV-1 and the ONT sequencing error render analysis of the generated data difficult. NanoHIV is a new tool that uses an iterative consensus generation approach to construct accurate, near full-length HIV-1 proviral single-genome sequences from ONT data. To validate the approach, single-genome sequences generated using NanoHIV consensus building were compared to Illumina^®^ consensus building of the same nine single-genome near full-length amplicons and an average agreement of 99.4% was found between the two sequencing approaches.

## 1. Introduction

As of 2019, 38 million people are living with HIV [1]. Although the introduction of early and effective antiretroviral therapy (ART) has led to significant declines in transmissions, morbidity, and mortality, HIV remains incurable in the large majority of individuals. Moreover, adherence challenges and the development of drug resistance threaten the long-term success of ART [2,3]. Therefore, investigating potential future HIV cures and developing and assessing the most durable therapies continue to be a priority for HIV research. Genomic characterization of HIV is an essential component of this research and relies on PCR followed by Sanger or next generation sequencing (NGS) [4,5]. Longitudinal characterization of single HIV genomes at limiting dilution enables one to investigate viral adaptation under immune and drug pressure and to monitor HIV persistence in long-lived and proliferating cell populations.

HIV infection in most susceptible cells leads to active replication with viral release and cell death. However, a small subset of infected cells persist and carry intact proviruses that are hidden from the immune response and unaffected by ART [6,7], known as the latent reservoir. The latent reservoir is the major barrier to an HIV cure in well-treated individuals. To provide an in-depth analysis of the latent reservoir, novel near full-length (NFL) proviral amplification and single-genome sequencing assays have been developed. These assays overcome the limitations of sub-genomic sequencing which do not account for large internal deletions or deleterious mutations outside of the target region [8,9,10,11,12]. NFL proviral sequencing involves limiting dilution of PCR using primers targeting the outermost gag leader and long-terminal repeat (LTR) regions of the viral genome resulting in ~9 kb fragments that are about 92% of the proviral genome. NGS via Illumina^®^ sequencing is the most established method and is followed by the assembly of sequence reads to a genetically intact reference to assess the presence of frameshifts, inversions, premature stop codons or large internal deletions that could render these proviruses defective [10,11,12,13].

A high through-put, efficient and standardized bioinformatic pipeline that assembles full-length proviral genomes is vital to increasing our understanding of the genomic composition and dynamics of infected cells that persist during ART. For instance, little is known about the correlation between immune escape variants amongst integrated, sequence-intact proviruses and circulating plasma viruses [14]. Generating long-read viral envelope sequences would also enable the assessment of viral diversity, cellular tropism and escape from neutralizing antibodies, especially in underrepresented populations with a high disease burden [15,16]. In addition, long sequence reads that include the variable loops of the envelope gene would provide sensitive detection of viral evolution and compartmentalization in central nervous system (CNS) and other tissues [17,18,19].

New evidence suggests that ART resistance may be conferred by HIV mutations outside the drug target gene. For example, mutations located in the polypurine tract (PPT) [20] and in the gp120 and gp41 interface [21] can confer resistance to dolutegravir, an HIV-1 integrase strand transfer inhibitor (INSTIs). Investigating these mechanisms in ART-treated cohorts requires long-read sequencing that should be both cost-effective and scalable.

The ability to generate long-read lengths has largely been facilitated by third-generation sequencing technologies such as those developed by Pacific Biosciences (PacBio) and Oxford Nanopore Technologies (ONT). These sequencing approaches have overcome various shortcomings observed in Sanger and Illumina^®^ sequencing. Whereas Sanger sequencing offers a fast-sequencing method for low numbers of targets, second-generation sequencing, or NGS, including Illumina^®^, offers massively parallel, accurate and cost-effective sequencing. However, the most significant shortcoming of both Sanger and Illumina^®^ sequencing is the short-read lengths of up to 1000 bp [22]. Despite these methods being relatively accurate, the generation of short reads make resolving repetitive regions in genomes difficult [23,24,25]. Third-generation sequencing offers unique approaches and chemistries enabling long-read (>10 kb) sequencing of nucleic acid molecules in real-time and at high resolution. Moreover, these technologies could preserve base modifications when sequencing native DNA thereby avoiding the bias introduced by primers utilized in Sanger sequencing. Long read sequencing also improves *de novo* assemblies of complex genomes including repetitive regions, improves mapping certainty and enables the detection of structural variants [22,23,24,25,26].

Major technical differences separate PacBio’s single-molecule, real-time (SMRT) sequencing technology and ONT’s nanopore sequencing. SMRT sequencing derives the nucleotide sequence by detecting fluorescence events that correspond to the incorporation of four fluorescently-labelled nucleotides by a DNA polymerase that is affixed within a zero-mode waveguide (ZWM) on a SMRT Cell [27,28]. SMRT is often the preferred third-generation sequencing method due to the reported lower error rate [24,25]. The SMRTbell library preparation, which involves the ligation of hairpin adaptors to the ends of double-stranded DNA fragments, allows the circular DNA library template to be re-sequenced multiple times to increase read accuracy through circular consensus sequencing (CCS). However, the read length and number of passes of the SMRTbell library are limited by the processivity of the DNA polymerase enzyme that is utilized in the sequencing reaction and, therefore, shorter library inserts are preferentially sequenced [24]. In addition, CCS reads retain errors and exhibit a bias for insertions and deletions in homopolymer regions [25].

In contrast, ONT sequencing measures the changes in ionic current that occur across an electrically resistant polymer membrane as single-stranded nucleic acids pass through biological nanopores that are present on a flow cell. The nucleic acid sequence is inferred as resistance is dependent on which nucleotide bases occupy and surround the pore which is measured by an arrayed sensor chip and passed to an Application-Specific Integrated Circuit (ASIC) [25,29]. ONT sequencing provides the longest read lengths of all sequencing technologies, with library inserts of 10,000 to 30,000 bp commonly reported and the most recent record read length being 43 Gb [30]. Despite the long-read length capabilities; insertions, deletions and substitutions are frequently observed in ONT sequencing data which is influenced by the biological nanopore present on the flow cell. Resolving low-complexity stretches and homopolymer sequences is difficult as the current that is measured is a function of the particular k-mer that resides in the nanopore at the time and, because translocation of homopolymers does not change the sequence of the nucleotides within the pore, it results in a constant signal that makes determining homopolymer length difficult. To overcome this difficulty, ONT has developed novel R10 pores which differ from prior R9 pores in having a longer barrel and dual reader head which improves read accuracy over homopolymer regions substantially [31].

Data generated by conventional ONT displays a higher per-base error rate when compared to sequencing data generated by non-single-molecule sequencing such as Illumina^®^ [32] or IonTorrent [33,34]. The simplest, most cost-effective but also most error-prone mode on which to run an ONT sequencing reaction is the “1D” mode, where each strand of DNA is passed through the pore only once [25]. Raw signal generated by R9 and R10 pores is converted to a string of bases by the Guppy basecaller (v 2.2.2.1). However, both pore types display a raw per-base error rate of about 5% in 1D mode [31,35,36].

For well-conserved genomes (or genome regions) high read coverage alone could overcome most sequencing errors. Sequencing reads are first aligned to the reference genome using an ONT-specific reference mapping tool such as minimap2 [25]. A consensus sequence can then be produced by calling the majority base at each position with a tool such as samtools pileup [36]. This approach corrects random insertions and substitutions, but cannot entirely remove more systematic errors in ONT data, such as homopolymer errors [24,25,37].

HIV-1, however, has regions such as the variable loops in the envelope gene (Env) that are very poorly conserved across subtypes and even within subtypes and within donors [38,39]. In these regions, it is difficult or impossible to produce a high-quality alignment of an ONT read to a reference sequence using tools such as minimap2, because true variation, including insertions and deletions, is indistinguishable from sequencing errors, especially for homopolymer regions [40]. This problem can potentially be addressed by a *de novo* assembly approach using tools such as Canu [41]. *De novo* assembly does not require a reference genome and its accuracy is not affected by the intra-variant heterogeneity of HIV-1. However, this approach is computationally expensive and not guaranteed to succeed. *De novo* assembly is not an exact method, and early assumptions in de Bruijn graph formation caused by sequencing errors can result in the formation of false contigs [42].

These problems, while most significant in ONT data, are also present when sequencing HIV-1 data using other platforms. The tool SHIVER, designed to be used with data from the Illumina^®^ [43], solves this problem using a hybrid *de novo* assembly approach, where *de novo* contigs are aligned to create a draft consensus, to which the original sequencing reads are then aligned to refine the consensus, check its accuracy and remove false contigs. This approach requires several manual steps and is not ideal for high-throughput use.

Here, we present the new NanoHIV tool, a novel method for generating HIV-1 consensus sequences from ONT data. NanoHIV uses a bootstrap approach to refine a consensus sequence, including the refinement of variable regions, by first constructing a consensus sequence built from only highly conserved regions and then refining it by including variable regions from long reads as insertions.

## 2. Data Generation

### 2.1. Inclusion Criteria & Data Collection

Nine children from the Children with HIV Early Antiretroviral Therapy (CHER) cohort who initiated ART between ages 1.7 and 11.1 months were selected for investigation (Table 1). The participants were selected on the basis of having a total HIV-1 DNA count above 40 copies/10^6^ PBMC [44,45]. At the time of testing, the participants had been on ART for 6–9 years. Furthermore, these participants were selected based on the probability of obtaining intact proviral genomes. Previously, Katusiime et al. identified seven intact NFL proviral genomes from three of the nine children using Illumina^®^ MiSeq™ sequencing [12]. These seven intact genomes were sequenced in the current study with ONT. In addition, two HIV genomes known to be defective were included in the analysis. The therapeutic histories and the respective number of proviral genomes in the participants selected for ONT sequencing are shown below.

### 2.2. Near Full-Length Amplicon Generation

The methods described in Katusiime et al. [12] were used to generate amplicons for Illumina^®^ MiSeq™ and ONT sequencing. In brief, genomic DNA was extracted from peripheral blood mononuclear cells (PBMCs), diluted to a proviral endpoint and single HIV genomes were amplified with a nested near full-length (NFL) PCR using Ranger mix (Bioline, London, UK). The initial amplifications were performed using previously described primers with minor modifications to allow for HIV-1 subtype C amplification as shown in Table 2; the pre-nested primer set included Li_OuterF and Li_OuterR; the nested primer set used Li_InnerF and Li_InnerR [46] to generate amplicons of 8.8 kb. However, the protocol was later adapted as a hemi-nested PCR approach, where the first round PCR remained unchanged while the second round PCR was performed using a newly designed primer, NFL_alt_in_F [12] and Li_OuterR to amplify an important region of HIV that included the packaging signal (Table 2), as this region was recently shown to be essential for replication competence [47]. Amplicons resulting from the adapted NFL approach measuring approximately 9 kb were used for single-genome sequencing. 

### 2.3. MiSeq™ Library Preparation

MiSeq™ library preparation and sequencing were performed at the Institute for Microbial Biotechnology and Metagenomics (IMBM) at the University of Western Cape. The Illumina^®^ Nextera DNA library prep kit (Illumina, San Diego, CA, USA) was used as previously described [12]. In brief, DNA was enzymatically fragmented and adapters added to the template. The DNA was then purified and amplified by PCR that indexed the samples by adding different primer pairs to individual samples. The 300 cycle V2 MiSeq™ Reagent kit (Illumina, San Diego, CA, USA) was used to sequence the library. 

### 2.4. Bioinformatic Analysis of MiSeq Data

After sequencing, all reads with the same identifying index were assembled to form a consensus sequence. The sequences were then subjected to checks for viral intactness using the HIVIntact intactness pipeline [48]. Sequences were first checked for correct size (8.8 kb). Next, sequences that appeared to be mixed templates were detected and eliminated from further analysis. The remaining sequences were then translated to allow further analysis of the nine viral open reading frames (ORFs). A sequence was determined to be intact if, within these ORFs, there were no stop codons, frameshift mutations, hypermutations or deletions that could preclude viral infectivity.

### 2.5. Oxford Nanopore Technologies GridION Sequencing

Seven intact and two defective HIV-1 proviruses identified through the analysis of the Illumina^®^ MiSeq™ sequencing data [12] were selected for ONT sequencing. Using the high-fidelity Ranger Mix enzyme, the pre-nested PCR products corresponding to the identified intact or defective products were used to generate additional NFL amplicons for ONT sequencing [12].

### 2.6. ONT Library Preparation

The newly generated NFL amplicons were purified with AMPure XP paramagnetic beads (Beckman Coulter, Brea, CA, USA) (AMPure XP beads) using an optimized amplicon to bead ratio of 1:0.8 to ensure purification of products > 1.5 kb. Two wash steps were performed using freshly-prepared 80% ethanol and the bound DNA was eluted in 5 mM Tris-HCl. The purity and concentration of the purified NFL amplicons were measured using the NanoDrop™ 1000 Spectrophotometer (Thermo Scientific, Waltham, MA, USA).

The Amplicons by Ligation protocol (Oxford Nanopore Technologies, Oxford, UK, 2019) (ACDE_9064 _v109_revN_14Aug2019) provided by ONT was followed with minor modifications to prepare the DNA library for sequencing. Freshly-prepared 80% ethanol was used for the purification wash steps and the drying time of the bead pellet prior to elution was lengthened as needed until a change in appearance from shiny to matte was observed. Following the ligation of ONT’s sequencing adaptors, ONT’s Long Fragment Buffer was used for the final wash. The bead pellet was incubated at 37 °C for 10 min to increase DNA recovery of the longer NFL HIV fragments. The concentration and purity of the prepared DNA library was measured with the Qubit™ 2.0 Fluorometer and the NanoDrop™ 1000 Spectrophotometer, respectively. 

### 2.7. ONT Sequencing Conditions

FLO-MIN106D flow cells with R9.4.1 pores were primed using the reagents from the Flow Cell Priming Kit (EXP-FLP002) and following the instructions in ONT’s Amplicons by Ligation protocol. The final steps in the DNA library preparation were completed immediately before the prepared library was loaded into the SpotON port of the flow cell as described in the protocol. A new flow cell was used for each sequencing reaction, and a total of nine flow cells were used.

The following sequencing parameters were selected for all sequencing runs; DNA sequencing with SQK-LSK109, fast-basecalling, FAST5 and FASTQ files were selected as sequence data outputs and MinKNOW Release 19.10.2 or 19.12.2 software was used. The duration of the run was left as standard and the run was stopped when the sequencing throughput declined and sufficient nanopores were no longer available for successful sequencing.

## 3. Pipeline Description

The NanoHIV tool takes a folder of ONT FAST5 pore signal data from a single-molecule ONT HIV-1 experiment, and the resulting called FASTQ reads, as input. The FAST5 data are used to call variants and correct homopolymers, while the FASTQ reads are used for reference mapping.

**Figure 1:**
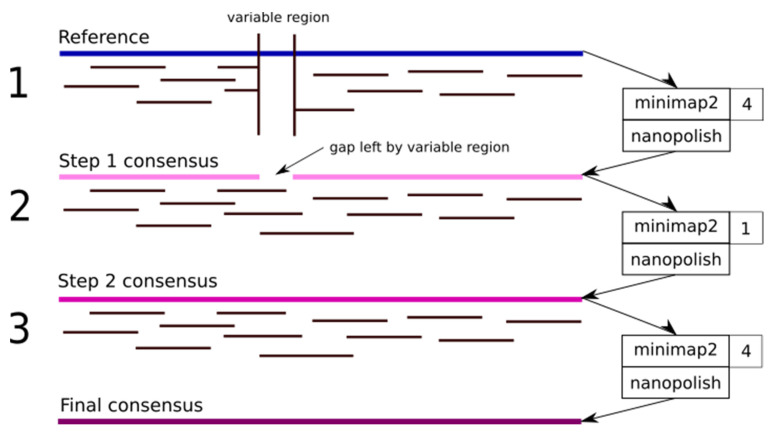
The HIV reference sequence contains many variable regions, particularly in the envelope gene (ENV), where conventional reference mapping may not be able to capture the high diversity. NanoHIV consists of three mapping steps: (**1**) An initial step with a standard gap penalty of 4, which results in a gap on the generated consensus where the variable region may be; (**2**) A remapping to the consensus with a lower gap penalty of 1, which bridges these variable regions; (**3**) A final mapping with the conventional gap penalty of 4 to remove any artefacts introduced by step 2.

The pipeline involves three mapping steps using minimap2 (v 2.17) [49] with different settings for each step (Figure 1). The samtools sorting and indexing functions are then used to sort the resulting SAM format files [50,51]. After each mapping step, the nanopolish consensus generation tool (v 0.11.3) is used to correct homopolymer errors and generate a file in the variant calling format (VCF) [52,53]. Finally, the nanopolish tool vcf2fasta is used to generate a final consensus sequence. In the first round of consensus generation, minimap2 is used with default settings. This step results in a consensus sequence where conserved regions represent the target DNA and non-conserved regions represent the consensus sequence. In this case, a subtype C consensus (GenBank ID AY772699.1) was chosen as a starting point, as this subtype is most likely to be detected in South Africa. The next round of consensus generation involves mapping the ONT reads to the consensus sequence generated in round 1, except with a gap opening and gap extension penalty 1/10th of the default setting. This round results in the long ONT reads spanning the HIV-1 variable loops (V1, V2, and V3) and other highly variable regions of the genome being aligned with a deletion of the entire region in the consensus sequence and an insertion of the entire region in the read. The final round of consensus generation involves taking the consensus sequence from round two and remapping the original ONT reads a third time. This final round corrects any additional false insertions or deletions created by round two.

## 4. Results

To validate our NanoHIV pipeline, we compared the HIV-1 consensus sequences generated from the 7 intact and 2 defective proviral genomes collected from four children in the CHER cohort using ONT and NanoHIV against consensus sequences generated from the same samples using an Illumina^®^ sequencer and the SHIVER pipeline. The mean similarity of mapped Illumina reads to the relevant Illumina consensus sequence was 98.9%, while the mean similarity of mapped ONT reads to the relevant consensus sequence was 92.1%. These figures are likely to be higher than the true similarity, paricularly for ONT reads, as they do not include very dissimilar reads rejected by the mapping algorithm.

In general, good agreement was found between the Illumina^®^ and ONT data. ONT sequences were slightly more similar to each other than Illumina^®^ sequences, both within and across donors. Pairs of Illumina^®^ sequences from the same donor were on average 99.0% similar, while pairs of ONT sequences from the same donor were on average 99.6% similar. Pairs of Illumina^®^ sequences from different donors were on average 90.8% similar, while pairs of ONT sequences from different donors were on average 91.6% similar. These findings suggest a bias in one or both pipelines. However, it cannot be easily determined whether ONT sequences are more similar due to too few high-quality variant bases being used to edit the original reference sequence, or whether artificial variants are introduced into Illumina^®^ sequences due to the *de novo* assembly step of the SHIVER pipeline. The genetic distance between ONT/Illumina^®^ pairs of sequences ranged from 98.6–99.7%, with a mean of 99.4% (Table 3). 

**Figure 2:**
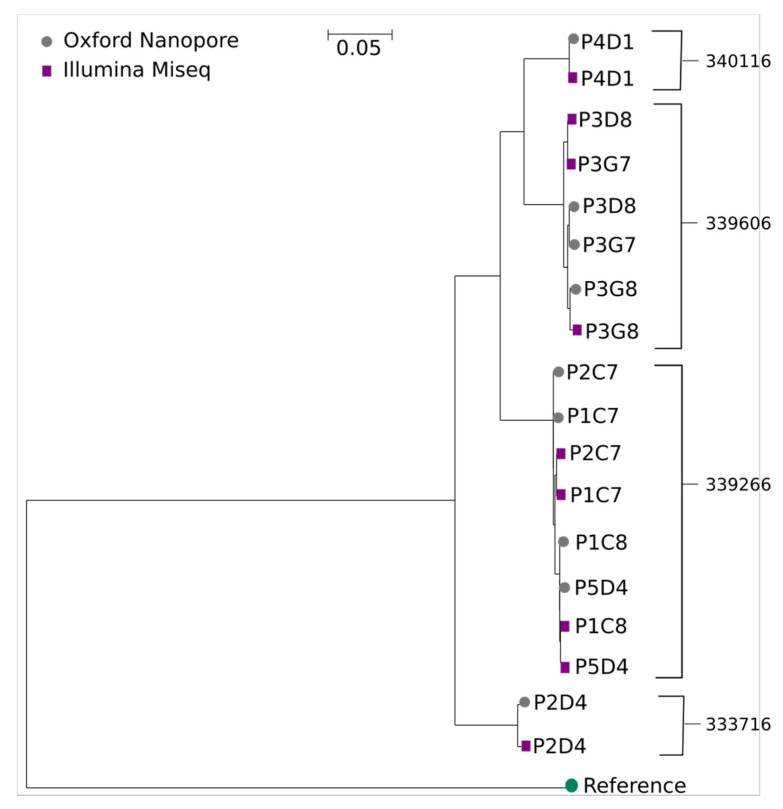
A neighbour-joining phylogenetic tree (generated using Dnapars from the PHYLIP package [54]) indicating the genetic distance between Illumina^®^ and ONT consensus sequences generated using SHIVER (Illumina^®^) and NanoHIV (ONT). There is generally good clustering between consensus sequences from the same sample but several very similar sequences from the same donor clustered more closely by pipeline than by sample, indicating potential artefacts in both pipelines. The tree is rooted on the subtype C reference (GenBank ID AY772699.1) used as the initial reference for the first round of NanoHIV.

We generated a phylogenetic tree from the sequence pairs and found good clustering in general. Several of the samples contained HIV genomes that were very close in genetic distance and, in these cases, the ONT sequences were likely to cluster closer together than the Illumina^®^ sequences (Figure 2). In particular, the P5D4 sample had coverage issues with the ONT method leaving too much of the original reference sequence in the final consensus. This failing, in turn, caused P5D4 to cluster closer to the P1C8 sample consensus than to its matching Illumina^®^ consensus.

## 5. Pipeline and Data Availability

The full pipeline has been implemented in Python 3, and can be run on any local machine or cluster that has minimap2 (v 2.17) [49], samtools (v 1.10) [50] and nanopolish (v 0.11.3) [52,53] in the path. The scripts are available for download via a GitHub repository at https://github.com/ramics/NanoHIV. The authors welcome contributions to the project.

The raw ONT sequencing reads have been uploaded to the NCBI Sequence Read Archive (https://www.ncbi.nlm.nih.gov/sra) and can be found at BioProject ID PRJNA765218.

## 6. Discussion

We have developed a new bioinformatics pipeline ‘NanoHIV’ which aims to overcome ONT sequencing error for highly variable genomes. Good agreement was found between ONT HIV-1 consensus sequences generated using NanoHIV and equivalent Illumina^®^ MiSeq™ sequences generated using SHIVER.

There are several advantages to ONT sequencing. It has the longest read length of all platforms. Three platform sizes offer flexibility of throughput, scalability and portability. Native DNA or RNA can be sequenced directly, providing epigenetic information. There are many library preparation methods including a convenient rapid 10-min library preparation method. The initial investment for ONT sequencing devices is less than PacBio’s SMRT sequencers. Furthermore, the sequencing cost per sample can be reduced by utilising a ‘read-until’ approach, where the sequencing run is terminated once sufficient coverage has been achieved. Sequencing costs can further be reduced by washing and reusing flow cells with sufficient active nanopores (≥800 as recommended by ONT) and multiplexing samples on a single flow cell. Oxford nanopore sequencing is the only real-time sequencing platform that allows for analysis without terminating the sequencing run, allowing for rapid diagnosis which could be useful in clinical and resource-limited settings [25,55,56]. Nevertheless, the high read error reduces the utility of ONT for variable genomes and requires novel solutions. Here, we present the development of a novel bioinformatics pipeline, NanoHIV, developed for and validated against highly variable HIV-1 single-genome sequences. 

The fact that ONT sequences were still likely to cluster more closely together than Illumina^®^ sequences in a phylogenetic analysis may indicate that too much of the original HIV-1 reference sequence is being included in each ONT consensus sequence, causing those sequences to appear more similar than they are in reality. It is also possible that a mirrored issue in the SHIVER pipeline caused the Illumina^®^ sequences to cluster too closely together.

NanoHIV was also assessed in an environment where the likely HIV-1 subtype, subtype C, was known, and an appropriate reference sequence could be chosen. Further work should be undertaken to ensure that NanoHIV is accurate when the HIV-1 subtype of the reference differs significantly from the subtype of the sequenced data.

Other approaches have recently been used to reduce ONT post-analytic error. For example, pre-sequencing circularization of amplicons followed by generation and sequencing of linear concatemers, an approach reminiscent SMRTbell library circularization, has shown 99.9% post-analytical sequencing accuracy of highly variable HIV quasispecies [57]. Moreover, new algorithms that take into account translocation time through pores may improve homopolymer length determination [58].

Additional improvements on NanoHIV could be considered in the future. A hybrid assembly step to scaffold insertions in the ONT consensus sequence, similar to the SHIVER approach, could remove any remaining reference bases in the consensus, which may expand the NanoHIV pipeline.

The NanoHIV pipeline has only been used on ONT data for HIV-1 to date, but is not limited to HIV-1 single-genome sequencing, and could be expanded to create single-molecule consensus sequences for other variable viruses, such as hepatitis C or influenza A viruses.

## Figures and Tables

**Table 1:** Study participant details, treatment histories and identified proviral genome status.

PID	Age ART Initiated (Months)	ART Regimen	Time on ART (Years)	No. of Proviral Genomes Sequenced with ONT	
				Identified as Intact	Identified as Defective
333716	2.3	AZT/3TC/LPV/r	8.55	0	1
339606	8.5	AZT/3TC/LPV/r	7.93	2	1
339266	9.23	AZT/3TC/LPV/r	8.2	4	0
340116	9.32	AZT/3TC/LPV/r	8.83	1	0

Therapy included Zidovudine (AZT), lamivudine (3TC) and lopinavir with low dose ritonavir (LPV/r).

**Table 2:** Near full-length single-genome amplification primers.

**Pre-Nested Primers**			
**Primer Name**	**Primer Direction**	**Nucleotide Position in HXB2 (bp)**	**5′-3′ Sequence**
Li_OuterF ^+,^*	Forward	623–649	AAATCTCTAGCAGTGGCGCCCGAACAG
Li_OuterR	Reverse	9662–9686	TGAGGGATCTCTAGTTACCAGAGTC
**Nested Primers**			
**Primer Name**	**Primer Direction**	**Nucleotide Position in HXB2 (bp)**	**5′-3′ Sequence**
Li_InnerF *	Forward	769–793	GCGGAGGCTAGAAGGAGAGAGATGG
Li_InnerR ^+^	Reverse	9604–9632	GCACTCAAGGCAAGCTTTATTGAGGCTTA
NFL-alt_in_F ^#^	Forward	642–664	CCG AAC AGG GAC BHG AAA GCG AA

^+^ Salminen et al., 1995, * Li et al., 2010, ^#^ Katusiime et al., 2020.

**Table 3:** Total aligned similarity between intact and defective HIV proviral genomes sequenced with Illumina^®^ MiSeq™ and ONT.

Patient Identifier	Sample Identifier	Proviral Genome Status	Total Aligned Similarity Percentage (%)
340116	P4D1	Intact	99.3
339606	P3D8	Intact	98.6
	P3G7	Intact	99.4
	P3G8	Defective	99.6
333716	P2D4	Defective	99.1
339266	P2C7	Intact	99.6
	P1C7	Intact	99.6
	P1C8	Intact	99.7
	P5D4	Intact	99.6

## Data Availability

The scripts are available for download via a GitHub repository at https://github.com/ramics/NanoHIV. The authors welcome contributions to the project.
